# Factors associated with nurses’ challenges in providing oral care at Oulu University Hospital, Finland

**DOI:** 10.2340/aos.v84.44806

**Published:** 2025-10-10

**Authors:** Roosa-Maria Kivilahti, Hannu Vähänikkilä, Marja-Liisa Laitala, Vuokko Anttonen, Anna-Maija Syrjälä

**Affiliations:** a Research Unit of Population Health, Faculty of Medicine, University of Oulu, Finland; b Northern Finland Birth Cohorts, Arctic Biobank, Infrastructure for Population Studies, Faculty of Medicine, University of Oulu, Finland; c Medical Research Center and Oulu University Hospital, Oulu, Finland

**Keywords:** Challenges in oral care, infection-sensitive patients, self-efficacy, oral health, university hospital

## Abstract

**Objective:**

To investigate factors associated with nurses’ challenges in providing oral care to infection-sensitive patients.

**Material and methods:**

A total of 114 nurses from four internal medicine wards and one oncology ward participated in the study. Data were collected using a questionnaire containing six items about challenges in providing oral care to patients. A multivariate linear regression model was used to analyze the association between explanatory variables and challenges in oral care.

**Results:**

Practical nurses reported more challenges in evaluating patients’ oral problems compared to registered nurses (B = 1.8, *p* ≤ 0.01). Nurses reporting fairly good or poor oral health reported more challenges in managing resisting patients than those who reported good oral health (B = 1.1, *p* < 0.05). Higher self-efficacy in *Practical skills* was associated with fewer challenges related to having a lack of knowledge about cleaning a patient’s mouth (B= −0.9, *p* ≤ 0.01) and evaluating patients’ oral problems (B = −0.5, *p* ≤ 0.01). Higher self-efficacy in *Confidence to detect oral problems* was associated with fewer challenges in managing resisting patients (B= −0.6, *p* ≤ 0.01) and evaluating patients’ oral problems (B = −0.5, *p* ≤ 0.001).

**Conclusions:**

Nursing education, self-perceived oral health, and self-efficacy were associated with perceived challenges.

## Introduction

A significant number of patients hospitalized with acute medical conditions experience compromised oral health [1]. During inpatient stays, oral health has been found to deteriorate due to decreased tooth brushing habits [2, 3], increased intake of a cariogenic diet [3] and an increase in plaque accumulation, incidence of mucositis, and gingival inflammation [4]. The dental plaque [5] of infection-sensitive patients has been found to be colonized by Gram-negative bacteria. This colonization increases the risk of bloodstream infections [6], respiratory and non-respiratory-related pneumonia [7], endocarditis, liver and lung infections [8], and other infection-related complications [9] among hospitalized infection-sensitive patients. Research has shown that professional oral care performed by dentists and dental hygienists [10], as well as daily oral care provided by nurses, significantly reduces the risk of pneumonia [10, 11] and perioperative infections in cancer patients [12].

Despite its proven importance, studies indicate that oral care is often neglected in routine nursing care in hospitals [13–16]. The challenges nurses face in providing oral care to patients may be one reason why it is neglected. Previous studies have shown that the major challenges nurses faced in oral care were time constraints [13, 15, 17], shortage of available methods or instruments [13, 18], lack of guidelines [13], and patients’ resistance to oral care [17–21]. On the other hand, Koike et al. [17] reported that nurses who had a strong interest in oral care and prioritized it, or had undergone prior experiential learning, were less likely to perceive oral care as a burden.

Previous research on nurses’ challenges in providing oral care [13, 15, 17–19] has not focused on nurses who treat infection-sensitive patients whose severe illnesses can be aggravated by oral infections. These patients, however, often have a reduced capacity to maintain good oral hygiene due to severe illness and its therapy. In our previous study, we reported good self-efficacy in practical skills related to oral care among nurses in university hospital inpatient wards where specialized medical care is offered to infection-sensitive patients [22]. In this study we further analyze nurses’ perceptions of oral care, and the specific objective was to investigate simultaneous factors associated with nurses’ oral care-related challenges when treating infection-sensitive patients.

## Materials and methods

### Study design, study participants, and settings

The study group of this questionnaire-based, cross-sectional study consisted of nurses recruited on four wards of internal medicine (cardiology, endocrinology and nephrology, pulmonology, and infectious diseases) and one ward of oncology (oncology and hematology) at the Oulu University Hospital, Finland. Typically, these university hospital wards treat infection-sensitive patients suffering from an acute and/or imbalanced disease that can be aggravated by oral infections; many of these patients also have a high risk of developing odontogenic infections. All the recruited nurses with different educational backgrounds (auxiliary nurses, practical nurses, and registered nurses) are equally responsible for patients’ oral care.

#### Data collection

The data for the study were collected in August 2020. In every selected ward a brief introduction was given before the start of the study. The purpose of the study was explained to the nurses, but no motivational aspects or information about patients’ oral care were included. Nurses were asked to complete an anonymous self-administered questionnaire. The questionnaires were collected in a closed box in each of the wards. Participation was voluntary, and the time given to respond was 2 weeks.

### Questionnaire

The questionnaire was originally developed for geriatric home care nurses and piloted in a previous study [23], and some modifications were made to make it more suitable for hospital settings.

### Outcome variables

#### Challenges in providing oral care

The questionnaire included six items about challenges experienced by nurses in providing oral care to patients in their daily nursing care (Table 1). Nurses answered the items using an 11-point Likert scale with a score of 0 for the answer *‘I fully disagree’* and a score of 10 for the answer *‘I fully agree’*. The challenge items were used as continuous variables in the analyses. The original questionnaire contained two almost similar challenge items, *‘I’m disgusted by a dirty mouth’* and *‘I’m disgusted by dirty dentures’,* of which the latter was excluded from the questionnaire.

**Table 1:** Descriptive statistics for outcome and explanatory variables (Mean, SD, *n*, %).

Study variables	
**Outcome variables**	
Challenging items in oral care (Mean, ± SD)	
I don’t have enough time, *n* = 114	6.0 (2.8)
Patients won’t let me to look in their mouth, *n* = 114	4.9 (2.5)
I can’t evaluate the problem in a patients mouth	4.3 (2.4)
I’m disgusted by a dirty mouth, *n* = 113	3.8 (3.6)
I don’t have enough knowledge how to clean a patient’s mouth	3.5 (2.7)
I don’t have the proper tools, *n* = 114	3.4 (3.1)
**Explanatory variables**	
Background variables *n* (%)	
Age, *n* = 114	
< 30	41 (36.0)
30–39	30 (26.3)
40–49	21 (18.4)
≥ 50	22 (19.3)
Basic education, *n* = 114	
Basic education of 9 years	3 (2.6)
Upper secondary education	19 (16.7)
Tertiary education	92 (80.7)
Education level in nursing, *n* = 107^1^	
Auxilary nurse	7 (6.1)
Practical nurse	12 (10.5)
Registered nurse	88 (77.2)
Years since graduation, *n* = 113	
< 5	46 (40.4)
5–9	26 (22.8)
10–14	11 (9.6)
> 14	30 (26.3)
Working experience in years, *n* = 112	
< 5	41 (36.0)
5–9	23 (20.2)
10–14	14 (12.3)
> 14	34 (29.8)
Nurses’ self-perceived oral health and oral health habits *n* (%)	
How would you describe your own oral health, *n* = 113	
Good	77 (67.5)
Fairly good	34 (29.8)
Poor	2 (1.8)
How often do you visit a dentist, *n* = 113	
For regular dental examinations	67 (58.8)
Irregularly	34 (29.8)
Just for a toothache or when I need other first aid	10 (8.8)
How often do you brush, *n* = 112	
At least 2 times a day	96 (84.2)
Once a day	16 (14.0)
More sheldom	0
How often do you floss, *n* = 112	
At least 2 times a day	10 (8.8)
Once a day	35 (30.7)
More sheldom	67 (58.8)
Self-efficacy factors in oral care (Mean, ± SD)^2^	
Self-confidence in taking care of patients’ oral hygiene, *n* = 113	6.2 (2.1)
Practical skills, *n* = 114	8.8 (1.1)
Confidence to detect oral problems, *n* = 114	4.9 (1.6)
Oral health-related knowledge scale (Mean, ± SD)	4.2 (0.5)

SD: standard deviation.

### Explanatory

#### Background variables

Participants were categorized according to their age (< 30 years/30–39 years/40–49 years/≥ 50 years), basic educational background (basic education of 9 years/upper secondary school/third level education), nursing education (auxiliary nurse/practical nurse/registered nurse), years since graduation (< 5 years/5–9 years/10–14 years/> 14 years), and working experience (< 5 years/5–9 years/10–14 years/ > 14 years). The differences in the scope of nursing education are explained in Appendix A. The categorized background factors were used in the analysis.

#### Nurses’ self-perceived oral health and oral health-related habits

Participants’ self-perceived oral health and their oral health-related habits were asked with the questions: *‘How would you describe your own oral health’* (good/fairly good/poor), *‘How often do you visit dentist’* (On regular examinations/irregularly/just for a toothache or when I need first aid), *‘How often do you brush your teeth’* (at least twice a day/once a day/more seldom). *‘How often do you floss’* (at least twice a day/once a day/ more seldom). Categorized variables related to self- perceived oral health and oral health habits were used in the analyses.

#### Oral health-related knowledge

*The questionnaire included nine items about nurses’ knowledge of oral health, and its* association with general health. The nurses’ oral health-related knowledge scale was described in our previous study [22]. The statements were *‘I have enough knowledge about oral health’, ‘Oral health affects general health’, ‘Bleeding from the gums when brushing is normal’, ‘Halitosis can be caused by bacteria in the mouth’*, *‘Poor oral health increases risk of aspiration pneumonia’*, *‘Some drugs reduce salivation’* , *‘Frequent consumption of sugar increases tooth decay’, ’Chronic oral inflammation can increase the risk of dementia’* and *‘Tooth loss is part of normal aging’.* Nurses answered the items using a five-point Likert scale (completely disagree/disagree/no idea/agree/completely agree). The items were scored on a scale from 1 to 5; a correct answer scored a point of 5, and a wrong answer scored a point of 1. A sum variable was formed, and its mean score was calculated. The mean score for the oral health-related knowledge scale was used in the analysis. Cronbach’s alpha was 0.636 for the oral health knowledge scale.

#### Oral health-related self-efficacy

The oral health-related self-efficacy scale was described in our previous study [22] and the scale was rated on an 11-point scale (0 = I am not at all confident, 10 = I am completely confident). Factor analysis of the scale in our previous study revealed three factors, Factor 1: *Self confidence in taking care of patients’ oral hygiene*, Factor 2: *Practical skills* and Factor 3: *Confidence to detect oral problems* (with corresponding Cronbach’s alphas for Factor 1: 0.797, Factor 2: 0.697 and Factor 3: 0.674). The items included in the factors are explained in Appendix B. The mean scores for factor scores 1–3 were used in the analysis.

### Statistics

The study population was described using frequencies and descriptive statistics according to background factors and variables related to nurses’ self-perceived oral health and their oral health habits. The challenging items in oral care and the sum scores of the knowledge scale and self-efficacy factor scores were described using means and standard deviations. The association between background variables (age, basic education, education level in nursing, years since graduation, working experience), self-perceived oral health and oral health habits, mean scores for the oral health-related knowledge scale and self-efficacy factor scores 1–3 as explanatory variables and challenges as outcome variables, was analyzed using pairwise comparisons with univariable linear regression analysis. Explanatory variables that were statistically significantly associated with outcome variables in the bivariate analysis were selected for multivariate adjusted linear regression analysis. As there was a correlation between the variables years since the graduation and working experience, working experience was selected for the multivariate model as a more significant background factor. Statistical analyses were carried out with SPSS version 25.0 (SPSS, Inc., Chicago, IL, USA).

### Ethical considerations

The survey was voluntary, and data were collected and analyzed without participants’ IDs. Permission from the medical superintendent of the Oulu University Hospital was obtained for the study, and The Ethical Committee of the Hospital District of Northern Ostrobothnia issued a positive statement for the study (date: 26.2.2020; study diary No: 31/2020).

## Results

A total of 114 nurses participated in the survey. Regarding education level 77.2% (*n* = 88) were registered nurses. As for working experience 36.0% (*n* = 41) of the nurses had less than 5 years, 29.8% (*n* = 34) had more than 14 years, while the rest had 5–14 years of working experience. Self-reported oral health was rated as good by 67.5% (*n* = 77) of the respondents and 84.2% (*n* = 96) reported brushing their teeth twice a day.

Mean scores for the perceived challenges in patient oral care varied from 3.4 to 6.0. The main challenge nurses had in providing oral care to patients was a shortage of time (mean 6.0, standard deviation [SD] 2.8), followed by the patients’ resistance to let the nurses look in their mouths (mean 4.9, SD 2.5), as well as insufficient competence to evaluate the patient’s oral problem (mean 4.3, SD 2.4). The least significant challenge was the lack of proper oral care equipment (mean 3.4, SD 3.1), as shown in Table 1.

Table 2 shows unadjusted associations between explanatory and outcome variables based on univariate linear regression analyses. The multivariate adjusted linear regression model (Table 3) showed that practical nurses were significantly more likely to experience challenges with the item *‘I can’t evaluate the patient’s oral problems’* compared to registered nurses. Nurses who reported their self-perceived oral health to be fairly good/poor had a higher likelihood of experiencing challenges with the item *‘Patients won’t let me look in their mouths’* than those who perceived their oral health to be good.

**Table 2:** Unadjusted associations between explanatory variables and challenges analyzed using linear regression analysis.

	Challenge 1	Challenge 2	Challenge 3	Challenge 4	Challenge 5	Challenge 6
	Unadjusted B, (95% CI)	Unadjusted B, (95% CI)	Unadjusted B, (95% CI)	Unadjusted B, (95% CI)	Unadjusted B, (95% CI)	Unadjusted B, (95% CI)
Background variables						
Age						
< 30	1.0	1.0	1.0	1.0	1.0	1.0
30–39	1.1 (−0.3 to 2.4)	0.3 (−1.0 to 1.6)	0.4 (−0.9 to 1.6)	1.6 (−0.2 to 3.3)	0.5 (−0.6 to 1.6)	0.1 (−1.3 to 1.6)
40–49	−0.3 (−1.8 to 1.2)	0.1 (−1.3 to 1.5)	−0.4 (−1.7 to 1.0)	1.0 (−1.0 to 2.9)	−0.5 (−1.8 to 0.7)	1.5 (−0.2 to 3.1)
≥ 50	−0.6 (−2.1 to 0.9)	−1.0 (−2.4 to 0.4)	−0.5 (−1.8 to 0.9)	1.1 (−0.8 to 3.0)	−1.2 (−2.4 to 0.0)	0.3 (−1.3 to 2.0)
Basic education						
Basic education of 9 years	−0.0 (−3.3 to 3.3)	0.8 (−2.4 to 3.9)	0.2 (−3.2 to 2.7)	1.1 (−3.1 to 5.3)	−1.3 (−4.0 to 1.5)	1.6 (−2.0 to 5.2)
Upper secondary education	−0.0 (−1.4 to 1.4)	−0.2 (−1.6 to 1.1)	−0.1 (−1.4 to 1.2)	1.3 (−0.5 to 3.1)	0.7 (−0.5 to 1.9)	−0.4 (−1.9 to 1.2)
Third level education	1.0	1.0	1.0	1.0	1.0	1.0
Education level in nursing						
Auxiliary nurse	−0.4 (−2.6 to 1.8)	−1.5 (−3.6 to 0.6)	−0.6 (−2.6 to 1.4)	−0.1 (−2.8 to 2.7)	−1.8 (−3.5 to 0.0)	−0.7 (−3.0 to 1.6)
Practical nurse	0.1 (−1.6 to 1.8)	0.3 (−1.3 to 2.0)	0.2 (−1.4 to 1.7)	2.6 (0.4 to 4.7)*	1.6 (0.2 to 3.0)*	0.6(−1.2 to 2.4)
Registered nurse	1.0	1.0	1.0	1.0	1.0	1.0
Years since graduation						
< 5	0.4 (−0.9 to 1.7)	1.1 (−0.1 to 2.3)	0.6 (−0.6 to 1.8)	−0.2 (−1.8 to 1.5)	2.1 (1.0 to 3.1)***	0.2 (−1.2 to 1.6)
5–9	0.1 (−1.4 to 1.6)	1.4 (−0.0 to 2.8)	0.5 (−0.9 to 1.8)	−0.9 (−2.8 to 1.0)	1.5 (0.3 to 2.6)*	−1.6 (−3.2 to 0.0)
10–14	2.3 (0.4 to 4.3)*	1.2 (−0.7 to 3.0)	0.2 (−1.6 to 2.0)	2.4 (−0.1 to 4.9)	2.2 (0.6 to 3.7)**	0.9 (−1.2 to 3.0)
> 14	1.0	1.0	1.0	1.0	1.0	1.0
Working experience						
< 5	−0.0 (−1.4 to 1.3)	0.8 (−0.4 to 2.0)	0.7 (0.5 to 1.8)	−0.7 (−2.4 to 1.0)	1.6 (0.5 to 2.7)**	−0.3 (−1.7 to 1.1)
5–9	0.1 (−1.4 to 1.7)	1.2 (−0.3 to 2.6)	−0.1 (−1.5 to 1.2)	−1.3 (−3.2 to 0.6)	1.6 (0.3 to 2.8)*	−1.1 (−2.8 to 0.5)
10–14	1.4 (−0.4 to 3.2)	1.1 (−0.6 to 2.8)	1.3 (−0.3 to 2.9)	1.2 (−1.1 to 3.5)	1.8 (0.4 to 3.3)*	−0.2 (−2.1 to 1.8)
> 14	1.0	1.0	1.0	1.0	1.0	1.0
Nurses’ self−perceived oral health and oral health habits						
How would you describe your own oral health						
Good	1.0	1.0	1.0	1.0	1.0	1.0
Fairly good/poor	0.4 (−0.8 to 1.5)	1.0 (−0.1 to 2.0)	0.9 (−0.1 to 1.9)	1.2 (−0.2 to 2.6)	1.6 (0.7 to 2.5)***	1.0 (−0.3 to 2.2)
How often do you visit dentist						
On regular examinations	1.0	1.0	1.0	1.0	1.0	1.0
Irregularly	−0.3 (−1.4 to 0.9)	−0.2 (−1.4 to 0.9)	0.7 (−0.6 to 1.7)	−0.0 (−1.5 to 1.5)	0.8 (−0.2 to 1.8)	−1.0 (−2.3 to 0.2)
Just for a toothache or whwn I need first aid	1.1 (−3.0 to 0.8)	0.0 (−1.8 to 1.8)	−0.2 (−1.9 to 1.5)	−1.2 (−3.7 to 1.2)	0.5 (−1.1 to 2.1)	−1.4 (−3.5 to 0.6)
How often do you brush						
At least 2 times a day	1.0	1.0	1.0	1.0	1.0	1.0
Once a day	0.4 (−1.2 to 1.9)	−0.2 (−1.7 to 1.2)	0.7 (−0.7 to 2.0)	−1.2 (−2.2 to 1.8)	0.7 (−0.6 to 1.9)	−1.3 (−2.9 to 0.4)
How often do you floss						
1–2 times a day	1.0	1.0	1.0	1.0	1.0	1.0
More seldom	−0.6 (−1.7 to 0.5)	1.2 (0.2 to 2.2)*	0.0 (−0.9 to 1.0)	−0.8 (−2.2 to 0.6)	0.7 (−0.2 to 1.6)	−0.3 (−1.5 to 0.9)
Self-efficacy factors in oral care						
Self-confidence in taking care of patients’ oral hygiene	−0.2 (−0.5 to 0.0)	−0.4 (−0.6 to −0.1)**	−0.1 (−0.3 to 0.1)	−0.1 (−0.4 to 0.3)	−0.4 (−0.6 to −0.2)***	0.0 (−0.3 to 0.3)
Practical skills	−0.2 (−0.7 to 0.3)	−0.8 (−1.3 to −0.4)***	0.1 (−0.3 to 0.5)	0.1 (−0.5 to 0.7)	−0.7 (−1.1 to −0.4)***	−0.5 (−1.0 to −0.0)*
Confidence to detect oral problems	−0.3 (−0.5 to 0.1)	−0.4 (−0.7 to −0.1)**	−0.4 (−0.7 to −0.1)**	0.1 (−0.3 to 0.5)	−0.7 (−0.9 to −0.4)***	0.1 (−0.3 to 0.4)
Oral health related knowledge scale	−0.5 (−1.7 to 0.7)	−1.1 (−2.2 to 0.1)	−0.1 ( −1.2 to 1.0)	0.9 (−0.6 to 2.4)	−0.7 (−1.7 to 0.4)	−0.0 (−1.3 to 1.3)

CI: confidence interval.

Challenge 1: I don’t have enough time; Challenge 2: I don’t have enough knowledge how to clean a patient’s mouth; Challenge 3: Patients won’t let me look in their mouth; Challenge 4: I’m disgusted by a dirty mouth; Challenge 5: I can’t evaluate the problem in a patient’s mouth; Challenge 6: I don’t have the proper tools.

* *p* < 0.05,

** *p* ≤ 0.01,

*** *p* ≤ 0.001.

**Table 3:** Multivariate adjusted linear regression models analyzing the association between explanatory variables and challenges.

	Challenge 1	Challenge 2	Challenge 3	Challenge 4	Challenge 5	Challenge 6
	Adjusted B, (95% CI)	Adjusted B, (95% CI)	Adjusted B, (95% CI)	Adjusted B, (95% CI)	Adjusted B, (95% CI)	Adjusted B, (95% CI)
Education level in nursing						
Auxiliary nurse	−0.7 (−3.2 to 1.8)	−0.5 (−2.7 to 1.7)	−0.9 (−3.0 to 1.2)	−0.7 (−3.8 to 2.5)	−0.8 (−2.5 to 0.8)	−1.2 (−3.9 to 1.4)
Practical nurse	−0.3 (−2.2 to 1.6)	0.5 (−1.2 to 2.2)	−0.4 (−2.0 to 1.2)	2.1 (−0.4 to 4.5)	1.8 (0.5 to 3.1) **	0.3 (−1.7 to 2.4)
Registered nurse	1.0	1.0	1.0	1.0	1.0	1.0
Working experience						
< 5	−0.5(−2.1 to 1.0)	0.2 (−1.1 to 1.6)	0.1 (−1.4 to 1.3)	−1.0 (−3.0 to 1.0)	0.8 (−0.2 to 1.9)	−0.3 (−2.0 to 1.3)
5–9	−0.4 (−2.2 to 1.3)	1.1 (−0.5 to 2.7)	−1.2 (−2.8 to 0.3)	−1.4 (−3.7 to 0.9)	0.9 (−0.3 to 2.1)	−0.9 (−2.8 to 1.0)
10–14	1.0 (−1.1 to 3.0)	0.4 (−1.4 to 2.3)	0.6 (−1.2 to 2.4)	0.3 (−2.4 to 3.0)	0.6 (−0.8 to 2.0)	−0.2 (−2.4 to 2.1)
> 14	1.0	1.0	1.0	1.0	1.0	1.0
How would you describe your own oral health						
Good	1.0	1.0	1.0	1.0	1.0	1.0
Fairly good/poor	0.4 (−0.9 to 1.6)	0.3 (−0.8 to 1.4)	1.1 (0.0 to 2.2)*	1.4 (−0.2 to 3.0)	0.8 (−0.0 to 1.7)	1.0 (−0.3 to 2.4)
How often do you floss						
1–2 times a day	1.0	1.0	1.0	1.0	1.0	1.0
More seldom	−0.9 (−2.1 to 0.4)	0.9 (−0.2 to 1.9)	−0.6 (−1.6 to 0.4)	−0.8 (−2.3 to 0.8)	0.1 (−0.7 to 0.9)	−0.2 (−1.5 to 1.1)
Self-efficacy factors in oral care						
Self-confidence in taking care of patients’ oral hygiene	−0.1 (−0.5 to 0.2)	−0.1 (−0.4 to 0.2)	0.1 (−0.1 to 0.4)	−0.1 (−0.5 to 0.3)	−0.0 (−0.2 to 0.2)	0.1 (−0.3 to 0.4)
Practical skills	−0.2 (−0.8 to 0.5)	−0.9 (−1.4 to −0.3) **	0.2 (−0.3 to 0.7)	0.0 (−0.8 to 0.8)	−0.5 (−1.0 to −0.1)) **	−0.5 (−1.2 to 0.1)
Confidence to detect oral problems	−0.1 (−0.5 to 0.3)	−0.1 (−0.5 to 0.3)	−0.6 (−1.0 to −0.2) **	−0.0 (−0.6 to 0.5)	−0.5 (−0.8 to −0.2) ***	0.1 (−0.4 to 0.5)

CI: confidence interval.

Challenge 1: I don’t have enough time; Challenge 2: I don’t have enough knowledge how to clean a patient’s mouth; Challenge 3: Patients won’t let me look in their mouth; Challenge 4: I’m disgusted by a dirty mouth; Challenge 5: I can’t evaluate the problem in a patient’s mouth; Challenge 6: I don’t have the proper tools.

* *p* < 0.05,

** *p* ≤ 0.01,

*** *p* ≤ 0.001.

Nurses who had higher self-efficacy regarding the factor *Practical skills* had a lower likelihood of experiencing challenges with the items *‘I don’t have enough knowledge to clean patient’s mouths’* and *‘I can’t evaluate the patient’s oral problems’*. Nurses who had higher self-efficacy on the factor *Confidence to detect oral problems* had lower a likelihood of experiencing challenge with the items *‘Patients won’t let me look in their mouths*’ and *‘I can’t evaluate the patient’s oral problems’.*

## Discussion

Lack of time, resisting patients and nurses’ perceived incompetence in evaluating oral problems were the nurses’ main challenges in providing oral care among infection-sensitive patients. Nursing education was related to experiencing challenges in evaluating patients’ oral problems, and nurses’ self-perceived oral health was related to patients’ resistance to let the nurses look into their mouths. Importantly, higher oral health-related self-efficacy was associated with fewer challenges related to patients’ resistance to having a nurse look in their mouth, incompetence in evaluating oral problems, and lack of knowledge of how to clean patient’s mouth.

Our results are in accordance with previous studies where nurses reported that limited time [13, 15, 17] is the main challenge in oral care, whether they work in an outpatient ward [17], generalized hospital [13] or acute care [15]. The lack of time for oral care is a significant problem that leads to a decline in oral hygiene and ineffective removal of dental biofilm among patients. In fact, it has been found that oral health deteriorates during hospital care, with decreased tooth brushing habit [2, 3], increased dietary intake of cariogenic foods [3] and an increase in plaque accumulation, incidence of mucositis, and gingival inflammation [4]. This impaired oral health is an important risk factor for infection-sensitive patients, predisposing them to bacteremia originating from the oral cavity, which has been found to lead to distant infections such as bloodstream infections [6], infective endocarditis, and liver and lung infections [8] among immunocompromised patients. A comprehensive health policy is needed to increase resources for health care in hospitals, thus enabling time for oral care and reducing the challenges of providing it, ultimately improving the oral health of patients.

In our study, patients’ resistance to let nurses look into their mouths was also found to be one of the major challenges. This result is in line with previous studies [17–21] where patient resistance to oral care was experienced as a significant challenge. Specifically, behaviors such as turning the head away or closing the mouth have been described as reactions to fear or cognitive impairment [18, 24]. To overcome these situations and improve oral care among resisting patients, guidelines called ‘Mouth Care Without a Battle’ have been published [21]. They offer an evidence-based practical approach to person-centered daily mouth care for persons with cognitive and physical impairment. In those instructions, according to the guidelines for providing oral care when a patient refuses to open his/her mouth, the nurse should be patient, try small talk and give reason for performing for oral care. The nurse may also show techniques of oral care to improve understanding of oral hygiene procedures. It is also possible to come back another time, when the patient may be more cooperative. On the other hand, all patients have autonomy, which means that the patients have the right to decide on their treatment and refuse a particular therapy, including oral care. Therefore, nurses face ethical issues when caring for patients who resist oral care.

We found no association between nurses’ working experience and perceived challenges in oral care. This aligns with findings from a study by Koike, where nurses’ clinical experience was not linked to perceiving oral care as a burden [17]. All nurses involved here with different educational backgrounds are responsible for patients’ oral care, and there is no task specification for patients’ oral care between the nurses. However, in our study registered nurses had less challenges in evaluating the problem in a patient’s mouth than staff nurses. In fact, according to our knowledge, the nursing programs for these degrees in Finland include limited or no oral care education [25, 26]. Thus, registered nurses’ perception of a lower level of challenge in evaluating oral problems in patients cannot be explained by nursing education, but can be explained, for example, by wider scope in the nursing field and greater responsibility in nursing care.

Nurses reported that the lack of knowledge about cleaning patients’ mouths was the second least challenging issue in oral care and oral health-related knowledge scale was not found to be associated with any challenges in oral care in the bivariate analysis. Our findings are in contrast with previous studies by Dagnew et al., who found that a lack of knowledge is a significant barrier for nurses in providing oral care [13]. Similarly, Koike et al. found that nurses who had learned about oral care through methods such as nursing school classes, training sessions, or seminars tended not to perceive oral care as a challenge [17]. The differences between previous studies and our study with regard to oral health-related knowledge as a barrier to oral care may be due to differences in the measurement of oral health-related knowledge and challenges, as well as the oral health-related education received and cultural and institutional factors.

Nurses whose self-perceived oral health was fairly good/poor were more likely to experience challenges with resisting patients than those whose oral health was good. As far as we know, there are no previous studies about the impact of nurses’ own oral care on their perceived challenges in delivering oral care to patients. However, in a study by Ashkenazi et al., a positive correlation was found between nurses’ personal oral hygiene habits and their commitment to maintain good oral hygiene for their patients [27]. The paucity of studies indicates a need for further investigation and an understanding of the association between nurses’ own oral health habits and their perceived challenges in providing daily oral care for patients.

We also found an association between nurses’ oral health-related self-efficacy and perceived challenges in providing oral care. The idea of self-efficacy is that people’s belief in themselves and their own abilities affects their actions and performance, and self-efficacy is an individual’s belief in how he/she manages to cope with challenges related to certain tasks [28]. Our study supports the theory of self-efficacy, as better self-efficacy in the factor *Practical skills* was associated with fewer challenges in evaluating the problem in a patient’s mouth and not having enough knowledge on how to clean a patient’s mouth. Furthermore, better self-efficacy in the factor *Confidence to detect oral problems* was associated with fewer challenges involving patients refusing to open their mouth and with evaluating the problem in a patient’s mouth. These results suggest that enhancing nurses’ self-efficacy in oral care, for example by targeted intervention and support, could potentially reduce those identified challenges. Altogether, oral health-related self-efficacy among nurses seems to be important and is associated with fewer challenges in providing daily oral hygiene for infection-sensitive patients.

### Strengths and weaknesses of the research

The reliability and validity of the challenge scale have been described earlier in a pilot study [23]. We analyzed our data using a multivariate regression analysis, which increases the reliability of the results, as we were able to control the effects of several simultaneous explanatory variables. Our structured questionnaire was clearly designed, which helps to minimize ambiguousness when answering the questions. While the structured questions in the questionnaire made the use of a multivariate analysis possible, the use of closed questions presents a limitation, as the fixed response options restrict the depth and nuance of the answers to the items. The scope of this study was confined to a few university hospital wards, thereby limiting the general applicability of the results to other hospitals. The study involved 114 nurses, with a noted limitation being the inability to accurately assess the response rate and to estimate the possible sampling bias due to varying numbers of staff (including substitutes for those on leave and individuals working simultaneously across different wards). Sending the survey to nurses by email could have been useful, and the response rate would have been analyzed. However, considering the nature of nurses’ work, nurses may not have enough time to respond to email survey during shift, and collecting paper questionnaires was considered more practical than digital ones, as the forms were easily accessible. As the answering to the questionnaire was voluntary, it is possible that participating nurses consider the topic more important when compared to dropouts. Social desirability in answers may also have to be taken into account. The aforementioned facts may introduce a slight positive bias in the results. However, the anonymity of the questionnaire encouraged respondents to answer truthfully.

### Implications

Nurses play a critical role in the oral care of infection-sensitive patients, and nursing education should include more comprehensive training on oral care and strategies for managing challenging patients. Treating resistant patients was identified as a major challenge, suggesting that training should focus on this area in particular. Based on the results, targeted training programs could be developed to improve nurses’ practical skills and self-efficacy in oral care. Since time constraints were the most significant challenge, increasing resources for health care is essential. For instance, in a recent study by Li et al., high workloads and a lack of nursing resources were major barriers to delivering oral care in an intensive care unit, and oral care was frequently deprioritized in the presence of more urgent tasks [18]. Thus, health care systems should prioritize time management and implement changes in the working environment to allocate more time for patient oral care. In conclusion, emphasizing oral care among infection-sensitive patients is crucial to improving their overall health.

## Conclusions

It can be concluded that the most significant challenges nurses face in providing oral care to patients in university hospital wards for infection-sensitive patients are a shortage of time and patients’ resistance to let nurses look in their mouth. Nurses’ educational level, nurses’ self-perceived oral health, and as a new interesting finding, nurses’ self-efficacy in patient oral care were associated with perceived challenges.

## Supplementary Material

Factors associated with nurses’ challenges in providing oral care at Oulu University Hospital, Finland

## Data Availability

The data supporting the findings of this study are available from the corresponding author [R-MK] upon reasonable request.
