# Supplementary material for: Factors associated with nurses’ challenges in providing oral care at Oulu University Hospital, Finland
Source: Acta Odontol Scand. 2025 Oct 10;84:44806. doi: 10.2340/aos.v84.44806 (PMC12519886; doi:10.2340/aos.v84.44806)
Supplement: Factors associated with nurses’ challenges in providing oral care at Oulu University Hospital, Finland [file AOS-84-44806-s1.pdf]

Supplementary material has been published as submitted. It has not been copyedited or typeset by Acta Odontologica Scandinavica.

Appendices:

Appendix A: Level of education among auxiliary nurses, practical nurses, and registered nurses

In Finland, the education of registered nurses is equivalent to a bachelor's degree and takes about 3.5 years. The education of a practical nurse lasts about 2-3 years in Finland, and it provides training for practice-oriented nursing tasks. Although auxiliary nurses, with the narrowest scope of training, are no longer formally educated, they still operate in the nursing field.

Appendix B: Questions included in self-efficacy factors

Factor 1 included items: *'I am confident that I brush patient's teeth, even when pressed for time'*, *'I am confident that I clean the patient's mouth, even when co-operation with him or her is problematic'* and *'I am confident that if a patient is unable to take care of their oral hygiene themselves, I look at their mouth every day'*. Factor 2 Included items *'I am confident that I know how to brush patient's teeth'* and *'I am confident that I know how to clean a patient's removable dentures*. Factor 3 included items *'I am confident that I am able to notice cavities in a patient's teeth'*, *'I am confident that I am able to notice inflammation of the mouth'* and *'I am confident that I am able to notice mucosal inflammation or ulceration related to a patient's use of removable dentures.*
